# Interventional cardiac catheterization predictors at Al-Arabi heart Center in Palestine in 2017

**DOI:** 10.1186/s12872-019-1222-0

**Published:** 2019-10-28

**Authors:** Abdulsalam Alkaiyat, Reham Abumadi, Shuruq Atari, Wasef Sayeh, Hamzeh Al Zabadi, Zaid Sarawan, Fekri Bisharat, Nizar Shakhshir

**Affiliations:** 1 Faculty of Medicine and Health Sciences, An-Najah National University, P. O. Box 7, Nablus, Palestine; 2 Swiss Tropical and Public Health Institute, University of Basel, Socinstrasse 57, 4002 Basel, Switzerland; 3 Specialized Arab Hospital, Nablus, Palestine; 4 Joint MD Program, Palestine Polytechnic University, Hebron, Palestine

**Keywords:** Cardiac catheterization, Troponin, ECG, Interventional, Palestine

## Abstract

**Background:**

Cardiac catheterization is performed for both therapeutic and diagnostic reasons, in which the outcome may vary from only medical treatment to the need of percutaneous coronary intervention and ending with coronary artery bypass graft. The primary goal of this study was to determine predictors of revascularization.

**Methods:**

A retrospective cohort study was conducted on data collected from records of patients who underwent cardiac catheterization at Al-Arabi Heart Center in Palestine in 2017. Multivariate logistic regression analysis was carried out to assess the association of sociodemographic and pre-catheterization clinical predictors with revascularization.

**Results:**

A total of 1550 patients were included in the study. The participants mean age was 58 with a SD of 11.7 years, 73.6% were males. 50.2% of patients who underwent an interventional cardiac catheterization tested negative for troponin on presentation. Multivariate logistic regression showed Troponin (RR = 4.5), Age (RR = 1.0), Female gender (RR = 0.4) previous catheterization (RR = 2.0), and existence of diabetes as significant predictors for revascularization. The correlation between ECG on presentation and the subsequent need for an interventional cardiac catheterization was significant only in case of ST-Elevation (RR = 1.5), and T wave inversion (RR = 1.6). CK-MB, Hypertension and ECG with ST-depression were not significant predictors.

**Conclusion:**

This study assessed revascularization predictors in addition to characteristics and outcomes of patients who have undergone cardiac catheterization. The results showed the especially high predictive value of troponin in determining the need for revascularization which outweighed the importance of ECG findings on presentation in making clinical decision regarding catheterization.

## Background

Coronary heart disease (CHD) remains the leading cause of death in Palestine accounted for 30.6% of total deaths recorded in 2016 [1]. Cardiac catheterization is performed for diagnostic reasons of CHD, and for revascularization which is achieved by PCI. It can also be achieved by Coronary Artery Bypass Graft (CABG) when catheterization is not applicable [2, 3]. The decision to undertake the catheterization should be based on the risks and benefits. It might be used as an emergency procedure to treat a heart attack, open up blocked arteries and prevent further heart damage.

The risk stratification in acute coronary syndrome (ACS) is usually based on the clinical data obtained during hospitalization [4]. Classical considerations for risk stratification are History, ECG, Age, Risk factors (smoking, hyperlipidemia, obesity) and Troponin [5]. Cardiac-specific monoclonal antibodies to cardiac troponin I (TnI) and troponin T (TnT) [6], along with the patient’s history, physical examination and electrocardiography are collectively used to predict the need for the most appropriate management plan in ACS.

Early diagnosis and intervention can be crucial in the management of patients with ACS. There are several tools that can lead to the right diagnosis. The presence of typical chest pain is considered one of them. However, its absence cannot rule out ACS as in patients with diabetes, hypertension or elderly patients. ECG is another important tool that is helpful in making the diagnosis of ACS, but it lacks sensitivity since 30–50% of patients may not have any diagnostic changes in their ECG very early during their presentation [7].

This retrospective cohort study examines risk factors (age, gender, past medical history including diabetes and hypertension, history of previous catheterization) to identify factors which might be correlated with the necessity of revascularization (PCI/CABG).

## Methods

### Study design and setting

A retrospective cohort study was conducted at the Heart Center in Specialized Arab Hospital in Nablus- Palestine. The study was conducted on data collected from records of 1550 patients who underwent cardiac catheterization in 2017. In this study, the significance level was set at a *p*-value = < 0.05, and a confidence interval at 95%.

### Inclusion and exclusion criteria

The candidates of this study were patients who underwent cardiac catheterization at Al-Arabi Hospital between January 2017 and December 2017. Patients who underwent valve replacement procedures were excluded from the study.

### Data collection tools

Selected sociodemographic and clinical characteristics were extracted from files of all patients who underwent cardiac catheterization in 2017at Al-Arabi Heart Center. Data extracted included specific risk factors (age, gender, past medical history including diabetes and hypertension, history of previous catheterization). Clinical Laboratory results for the included subjects were then recorded. Clinical tests included cardiac biomarkers (troponin and CK-MB). Furthermore, ECG findings obtained on presentation for each patient were categorized into: Normal or non-significant ECG findings, ST segment elevation, ST segment depression, T wave inversion, and other ECG findings.

Post catheterization outcomes were also documented and classified as: Normal coronaries for medical treatment, PCI including stenting or balloon, referral for CABG, this classification was the final outcome used for analysis.

### Statistical analysis

Data was entered, cleaned and managed using Microsoft Excel. Stata version 14 was used for data analysis. Categorical data was reported as frequencies, continuous data was reported as means. Bivariate analysis for the main outcome was conducted using Chi-square test or student t test when appropriate. Variables with significant *p* value were later analyzed in multivariate logistic regression model. Risk Ratio (RR) and confidence intervals are reported. Significant level is determined at p value ≤0.05.

## Results

A total of 1550 patients who underwent cardiac catheterization at AL-Arabi Heart Center in 2017 were included in this study. Sociodemographic and pre-catheterization clinical characteristics of patients were extracted including age, gender, and existence of chronic medical diseases such as: diabetes mellitus and hypertension, history of previous catheterization, ECG findings at presentation, and other related laboratory values such as: troponin, CK-MB.

### Sociodemographic and clinical characteristics

The mean age of the sample was 57.8, 73.6% were males, and 79.8% had one ECG change at least. 28,9 and 26.8% tested positive for Troponin and CK-MB respectively. Table 1 illustrates sociodemographic and clinical characteristics of the sample.

**Table 1:** Sociodemographic and pre - catheterization clinical predictive characteristics (*N* = 1550)

Patients’ Characteristics	N (%)
Mean Age (SD)	57.77 (11.68)
Male	1140 (73.55)
ECG (%)^1^	
Normal	294 (19.2)
ST elevation	436 (30.1)
ST depression	193 (12.6)
T wave inversion	279 (18.2)
Others	292 (19.0)
Missing	14 (0.9)
Previous catheterization (%) N = 1550	604 (39.0)
Diabetes mellitus (%)	627 (40.5)
Hypertension (%)	844 (54.5)
Outcome detailed (%)	
Normal/medical treatment	609 (39.3)
PCI	836 (53.9)
CABG	104 (6.7)
Missing	1 (0.1)
Troponin Positive^2^	353 (28.9)
CK-MB Positive	415 (26.8)
^1^N = 1536	
^2^ *N* = 1222	

### Predictors of revascularization

Table 2 compares between patients who underwent interventional and non-interventional cardiac catheterization in terms of sociodemographic and clinical characteristics. Age, sex were significantly different between patients who needed only medical treatment and those who needed revascularization. On the other hand, clinical characteristics such as ECG on presentation, previous catheterization and having either diabetes mellitus or hypertension were significantly different between patient who needed only medical treatment and those who needed revascularization. Among biomarkers, Troponin was significantly correlated with revascularization.

**Table 2:** Relationship between pre- catheterization predictive characteristics sociodemographic or clinical, and cardiac intervention (percutaneous coronary intervention/ coronary artery bypass graft)

	Intervention (*N* = 940)	No intervention (*N* = 610)	*P*-value
Mean Age in years (SD)	59.87 (10.7)	54.53 (12.3)	< 0.001
Gender			< 0.001
Male — N (%)	739 (64.8)	401 (35.2)	
Female — N (%)	209 (51.0)	201 (49.0)	
ECG (%)			< 0.001
Normal — N (%)	142 (47.8)	155 (52.2)	
St-elevation — N (%)	320 (68.7)	147 (31.3)	
ST-depression — N (%)	126 (64.6)	69 (35.4)	
T wave inversion — N (%)	175 (62.1)	107 (37.9)	
Others — N (%)	170 (57.6)	125 (42.4)	
Previous catheterization — N (%)	446 (73.8)	158 (26.2)	< 0.001
Diabetes mellitus — N (%)	420 (67.0)	208 (33.0)	< 0.001
Hypertension — N (%)	537 (63.6)	307 (36.4)	0.009
Troponin positive — N (%)	295 (83.6)	58 (16.4)	< 0.001
CK-MB — N (%)	253 (78.3)	70 (21.7)	< 0.001

After Multivariate logistic regression, Troponin, age, gender, previous catheterization and DM showed significant difference between patients who underwent an interventional cardiac catheterization and patients with no intervention after heart catheterization (p- value ≤0.05). CK-MB and Hypertension did not show significant difference. ECG finding at presentation was significant for ST-elevation and T wave inversion, but not in case of ST-depression and other ECG findings. The statistical significance of each variable for interventional cardiac catheterization, the risk ratio, *p*-value and confidence interval of each variable are listed in Table 3.

**Table 3:** Multivariate logistic regression of percutaneous coronary intervention and coronary artery bypass graft with sociodemographic and pre-catheterization clinical predictors

	RR^a^	*P*-Value	95% CI	
Positive Troponin	4.5	< 0.001	3.00	6.88
Positive CK-MB	1.3	0.197	0.87	1.97
Age	1.0	< 0.001	1.02	1.05
Female Gender	0.4	< 0.001	0.29	0.54
Previous Cath.	2.0	< 0.001	1.51	2.65
DM	1.4	0.033	1.02	1.81
HTN	1.3	0.095	0.96	1.68
ECG presentation				
St-elevation	1.5	0.046	1.01	2.15
ST-depression	1.6	0.066	0.97	2.50
T wave inversion	1.6	0.038	1.02	2.39
Others	0.9	0.681	0.61	1.39

^a^Adjusted risk ratio

## Discussion

The management of ACS can be achieved either by medical or invasive methods. Treatment guidelines always start with lifestyle changes that focus on diets, smoking cessation and weight control. Then comes the medical treatment which focuses on treating the underlying conditions that accelerates atherosclerosis as hypertension, dyslipidemia and diabetes. Medical treatment also consists of preventive treatments [8]. The treatment options also include revascularization by PCI or coronary artery bypass graft that are usually used in high-risk patients with acute coronary syndromes as an invasive strategy that provides the best outcome with a significant reduction in death and MI compared with an initial conservative strategy.

The most appropriate treatment depends on the patients’ clinical characteristics and their status in risk stratifications. However, sometimes choosing the most appropriate treatment can be unclear due to multiple factors that may affect treatment plan. The time between the onset of symptoms and the presentation to the medical the center, the presence of PCI capabilities in the facility, and the risks/benefits of using thrombolytics if needed, and other are important factors to consider upon taking medical decision [9]. Understanding such factors may facilitate physicians to choose which patients will benefit from revascularization and when.

Despite the high prevalence of CHD in Palestine, very limited research has investigated sociodemographic factors that may facilitate decision making in revascularization. This study is the first in Palestine to characterize the sociodemographic and clinical characteristic of 1550 patients who underwent cardiac catheterization.

The mean age in this study (57.8 years) is similar to findings of neighboring countries [10], and the same is applied for gender ratio. Furthermore, Demographic data for patients undergoing only diagnostic procedures (no intervention) and those undergoing revascularization were compared. 66.0% of those undergoing diagnostic procedure were males, whereas over three quarters of those who underwent revascularization were males, while in a study conducted in the US in 2010 the percentage of male patients in both diagnostic and interventional groups was a little bit lower (56, 67%, respectively) [11].

Other findings contradict other published research. For example, a substantial proportion of patients who underwent an interventional cardiac catheterization had diabetes mellitus and/or hypertension with a percentage of 67.0, 64.0% respectively. These proportions were different in the of Dehmer GJ et al. in the USA which showed percentages of 36.0%,82.0%, respectively [11].

Moreover, in this current study, it was concluded that ECG at presentation had a significant predictive value for undergoing a cardiac catheterization. Similarly, a study conducted by the Rochester Epidemiology Project in 2004 found that among patients discharged from the hospital with a diagnosis of chest pain of undetermined origin, those with an initial abnormal ECG were at higher risk for a subsequent adverse cardiac event [12]. Although ST segment deviation, mainly elevation, and T wave inversion were significant predictors in this study, normal ECG at presentation did not exclude the need for cardiac catheterization since a percentage of 19.0% of patients who needed a cardiac catheterization initially had a normal ECG at presentation as they had other clinical or lab findings at the time of presentation that required cardiac catheterization. Such findings need further research and indeed broader clinical criteria for diagnosis of CHD.

A significant relation between (elevation or depression) and the subsequent need for an interventional cardiac catheterization was also deduced from our results, where 69.0% of patients with ST elevation and 65.0% of patients with ST depression underwent an revascularization thereafter. This is consistent with a study conducted in Netherlands which suggested that in a patient with acute chest pain the ECG, using the ST-segment deviation direction, is of value to help accelerate decision-making by identifying patients most likely to profit from a rapid revascularization [13].

Creatine kinase (CK) and its MB isoenzyme (CK-MB) were the most commonly used serologic tests for the diagnosis of myocardial infarction prior to the widespread adoption of troponin which is now the biomarker of choice for the detection of cardiac injury since it is highly sensitive and specific for cardiac injury [14]. This is consistent with the results of this study which demonstrated that 52.8% of patients who underwent revascularization were CK-MB negative.

An increased troponin level regardless of CK-MB level identifies patients at higher risk for ACS than those with uniformly normal cardiac biomarker levels [15]. In this study, there was a significant positive predictive value for troponin regarding the need for revascularization since 83.6%of troponin positive patients required a revascularization afterward. However, a troponin negative value per se should not eliminate the consideration of a cardiac catheterization decision since 50.2% of patients who have undergone cardiac catheterization and needed an intervention were troponin negative at presentation. These results are congruous with the fact that serum cardiac markers obtained after 6 to 8 h of symptom duration are highly effective in diagnosing k and can be used more appropriately to make final clinical decisions than those obtained within 6 h of symptom onset [16]. These results should be taken into consideration in the evaluation of patients presenting with chest pain. It is worth mentioning that backward stepwise regression was carried out, and it did not produce different results from the multivariate logistic regression presented.

Clinical predictive significance of troponin and the clinical picture of the patient (signs and symptoms) for cardiac catheterization outweighed other predictors, including ECG findings, excluding ST-elevation and T wave inversion. In an American study conducted in 2001 by karras DJet al, it was stated however that “At no time should results of serum marker tests outweigh ECG findings or clinical assessment of the patient’s risk and stability” which may disagrees with the results of this study regarding the predictive strength of ECG, which showed that 47.8% of patients who subsequently needed revascularization initially had a normal ECG at presentation. Further research and validation should be conducted, but these results stress on the fact that the clinical presentation of the patient remains the mainstay of clinical decision whether to undergo a cardiac catheterization or not.

## Conclusion

This study assessed interventional cardiac catheterization predictors in addition to characteristics and outcomes of patients who have undergone cardiac catheterization. The results of this investigation showed that the clinical predictive significance of troponin and the clinical picture of the patient (signs and symptoms) for cardiac catheterization outweighed other predictors including ECG findings on presentation. Physicians should be alert enough to the clinical history and presenting symptoms rather than only depending on laboratory values and ECG interpretation particularly when they are equivocal.

Furthermore, the results of this study can be used by physicians and researchers to further establish evidence based guidelines that can be used in the clinical decision of carrying out cardiac catheterization procedures in order to decrease related cardiac morbidity and mortality. Nevertheless, future studies concerning the approach to patients presenting with cardiac symptoms are needed.

## Data Availability

The datasets used and/or analyzed during the current study are available from the corresponding author on reasonable request.

## Abbreviation

ACS: Acute coronary syndrome
AMI: Acute myocardial infarction
CABG: Coronary artery bypass graft
CHD: Coronary heart disease
CK: Creatine kinase
ECG: Electrocardiography
PCI: Percutaneous coronary intervention
TnI: Troponin I
TnT: Troponin T
